# Effects of a Nonviolent Communication-Based Empathy Education Program for Nursing Students: A Quasi-Experimental Pilot Study

**DOI:** 10.3390/nursrep12040080

**Published:** 2022-11-11

**Authors:** Jieun Sung, Youngran Kweon

**Affiliations:** 1 Department of Nursing, Nambu University, Gwangju 62271, Republic of Korea; 2 Department of Nursing, Chonnam National University, Gwangju 61469, Republic of Korea

**Keywords:** nursing student, empathy, self-esteem, interpersonal relationship, communication

## Abstract

This study aimed to develop and examine the effects of a nonviolent communication empathy education program, based on a cyclical empathy model of self-esteem, empathic ability, interpersonal relationships, and communication competency for nursing students. Participants were first-grade nursing students from two different colleges in Korea. There were 62 participants: 32 and 30 in the experimental and control groups, respectively. The experimental group received six sessions of 120 min each. The sessions were based on nonviolent communication content and utilized teaching methods such as lectures, presentations, personal activities, group activities, role plays, assignments, and reflective journals. The data were analyzed with percentage, χ²-test, Fisher’s Exact test, and a two-group independent means t-test using the SPSS 24.0 program. There were significant increases in self-esteem (t = 4.06, *p* < 0.001), empathic ability (t = 5.22, *p* < 0.001), interpersonal relationships (t = 5.14, *p* < 0.001), and communication competency (t = 5.27, *p* < 0.001) in the experimental group compared to the control group. Therefore, a nonviolent communication empathy education program based on the cyclical empathy model is expected to be useful for the humanities and social education in a nursing curriculum. Furthermore, it can improve human nursing competency based on multidimensional empathy in clinical settings.

## 1. Introduction

Nurses’ empathic ability is a core competency for building therapeutic relationships with patients and practicing nursing care [1]. From the patient’s perspective, empathy from nurses is an important criterion for determining service satisfaction [2]. Patients with empathetic nurses perceive that they are receiving good care [3]. Therefore, a nurse’s empathic ability as a therapeutic tool affects the quality of relationships with patients and contributes to the treatment process and outcome [4]. 

In addition, empathy is essential for nurses’ personal growth and self-care. When nurses lack empathy, they cannot accurately recognize their own thoughts and feelings [5]. This may cause problems with relationships between nurses and their colleagues and between nurses and patients [6]. When such problems persist, the individual’s psychological well-being and the quality and efficiency of their work performance may decrease [7]. A previous study [8] reported that nurses who lacked empathy had high job-stress, experienced increased burnout and had high turnover intentions. Therefore, it is essential to help nurses to enhance their empathy, both for themselves and their organizations. 

Empathic ability can be enhanced through continuous and repetitive training. Recent nursing education emphasizes the importance of interpersonal relationships, on the premise of self-understanding and empathy, via humanities and sociological education for nursing students [9,10,11]. Nursing students should have practical and important learning experiences through interactions with patients, caregivers, and healthcare professionals during clinical practicums [10]. For nursing students to deal more effectively with the various relationships they experience, it is necessary to offer education that can enhance their empathy. In addition, empathy is an essential ability for nursing students to understand the patients in their care as individual human beings and to interact with them respectfully [11,12].

Empathy develops sequentially and continuously through a sequence of stages and is revealed through interactions between cognitive, emotional, and communicative components [13]. Nonviolent communication (NVC) is a communication method that enables individuals to build qualitative interpersonal relationships through deep reflections and compassion for human beings [14]. NVC pursues changes in an individual’s perspective or thinking about human beings rather than simply training communication skills. In addition, NVC systematically illuminates characteristics occurring inside us or between individuals through the empathy process and attempts empathic communications reflecting the understanding and resonation of human beings [13]. Therefore, NVC is an effective method for enhancing multidimensional empathy [14].

Previous studies in South Korea have used NVC interventions in elementary school students, high school students, college students, office workers, and mothers receiving parental education [15]. Regarding overseas studies, a study involving parolees for substance abuse treatment [16] and another study involving prison inmates [17] have reported that NVC training improved self-esteem, empathy, interpersonal relationships, and communication competency in the participants. However, no NVC-based empathy enhancement education for nursing students has been attempted. This study aimed to develop and apply an NVC-based empathy education program for nursing students and to verify its effects. The findings can be used as primary data for developing educational strategies for realizing holistic nursing care and enhancing empathic ability in nursing students. 

### Aims and Hypothesis

This study aimed to investigate the effects of a NVC-based empathy enhancement education program on self-esteem, empathic ability, interpersonal relationships, and communication competency in nursing students.

The hypotheses were as follows:

**Hypothesis** **1.** *the self-esteem score will be higher in the experimental group using the NVC-based empathy education program compared to the control group.*

**Hypothesis** **2.** *the empathy ability score will be higher in the experimental group using the NVC-based empathy education program compared to the control group.*

**Hypothesis** **3.** *the interpersonal relationship score will be higher in the experimental group using the NVC-based empathy education program compared to the control group.*

**Hypothesis** **4.** *the communication competency score will be higher in the experimental group using the NVC-based empathy education program compared to the control group.*

## 2. Materials and Methods

### 2.1. Study Design and Participants

This is a pilot quasi-experimental study aimed at investigating the effects of NVC-based empathy education program on self-esteem, empathic ability, interpersonal relationships, and communication competency in nursing students. The study period was from 30 October to 31 December 2019. The experimental group was given six sessions of the NVC-based empathy education program, and the control group was provided with portable mini cards containing the principles of NVC and lists of feelings and desires. The participants were randomly assigned to the experimental and control groups by coin tossing. 

The participants in this study were first-year nursing students at a university located in G city, South Korea, who did not complete the “Interpersonal Relations and Communication” nursing curriculum course, had no experience in receiving empathy-related education, and understood the purpose of this study and agreed to participate in this study. 

To select participants, a notification was posted via the bulletin boards of the nursing department websites from 30 September to 25 October 2019, after obtaining permission from the heads of the nursing departments at two universities in South Korea. The nursing students at the two universities who expressed their intention to participate in this study were informed about the purpose and procedure of this study and provided written consent to participate. The participants were assigned to either an experimental group or a control group by coin tossing; when a picture or a number appeared in a coin toss, they were assigned to either the experimental group or control group, respectively.

**Figure 1:**
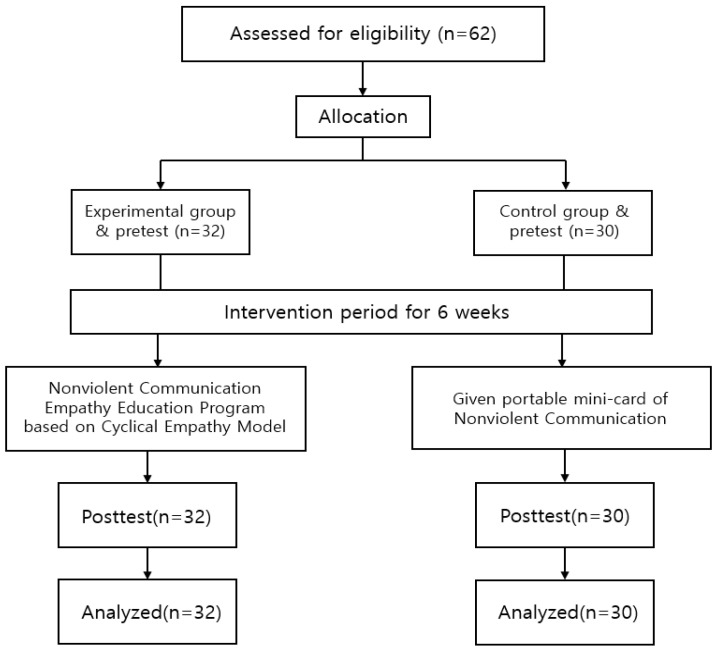
Participant selection flow.

The sample size required for this study was calculated using the G-power 3.1 program. The minimum number of participants for the two-group independent means t-test, with an effect size = 0.80 under the conditions of power (1-β) = 0.80 and a significance level (α) = 0.05, was calculated at a total of 52, with 26 participants each in the experimental and control groups (sample size ratio between group = 1:1). Considering a dropout rate, 62 participants were recruited, 32 in the experimental and 30 in the control group. However, there were no dropouts, and the final participants were 32 in the experimental group and 30 in the control group (Figure 1). The effect size in this study was calculated based on the results of a study using an empathy enhancement program in nursing students [18]. 

### 2.2. Instruments

In this study, all instruments were validated in Korean, and permission was obtained from the authors.

▪ Self-esteem was measured using the Korean version of the Rosenberg self-esteem scale, originally developed by Rosenberg [19] and translated into Korean and modified [20]. The scale comprises five items regarding positive, and five regarding negative, feelings about the self. Responses are rated on a 4-point Likert scale (1 = “strongly disagree”, 4 = “strongly agree”) and negatively-worded items are reverse scored. A higher score indicates higher self-esteem. The maximum score ranged from 10 points to 40 points. The reliability was Cronbach’s α = 0.80 in this study.
▪ Empathy ability was measured using the Korean version of the empathy scale, originally developed by Davis [21] and Bryant [22] and translated into Korean and modified [23]. This tool measures one’s cognitive ability to infer others’ psychological states and one’s ability to vicariously experience others’ emotions. It consists of 10 items regarding cognitive empathy and 20 items regarding emotional empathy. Responses are rated on a 5-point Likert scale (1 = “strongly disagree”, 5 = “strongly agree”) and negatively-worded items were reverse scored. The total score was then calculated. A higher score indicates a higher level of empathy. The reliability was Cronbach’s α = 0.83 in this study.
▪ Interpersonal relationship was measured using the Korean version of the interpersonal relationship scale, originally developed by Guerney [24] and translated into Korean and modified [25]. This tool measures the relationship between internal characteristics and external behaviors toward others. Responses are rated on a 5-point Likert scale (1 = “strongly disagree”, 5 = “strongly agree”) and the total score ranges from 25 to 125 points. A higher score indicates a higher interpersonal relationship. The reliability was Cronbach’s α = 0. 84 in this study.
▪ Communication competency was measured using the global interpersonal communication competence scale developed by Huh [26]. This tool measures appropriate and effective communication behavior in relationships with others. Responses are rated on a 5-point Likert scale and the total score ranges from 15 to 75 points. A higher score indicates better communication competency. The reliability was Cronbach’s α = 0.84 in this study.

### 2.3. Design of NVC-Based Empathy Enhancement Education Program

This study analyzed learner characteristics and interviews with clinical practitioners to provide empathy enhancement education needed for nursing students. First, to analyze learner characteristics, the nursing students’ nursing curriculum at two universities in G Metropolitan City was checked to investigate completed prerequisite subjects and clinical practicum. In addition, interviews were conducted with 4 nurses as clinical practitioners about situations requiring empathy in clinical settings, reasons empathy cannot be achieved, and what content would be needed for empathy education. The nurses had 4–10 years of clinical experience and, at the time interviews were conducted, were enrolled in the graduate school of nursing. In communication education for nursing students, various clinical cases are important, so 4 nurses working in internal medicine, surgery, psychiatry, and intensive care units were selected. They suggested that communication education is necessary for cooperative relationships with other occupations. However, they had no educational experience related to empathy or communication in clinical practice. In addition, regarding NVC-related literature [14,15], the educational contents were set to include all the cognitive, emotional, and communicative components of empathy [27]. Consistent with the process of empathy, the main contents were designed by dividing into empathic attention, empathic resonation, expressed empathy, perceived empathy, and the cycle of empathy. 

The contents of the NVC-based empathy education program by stage are shown in Table 1. Specifically, the 1st session was for participants to focus on others while reflecting on their communication style and observing others based on self-understanding, and participants practiced empathic attention. The 2nd and 3rd sessions were for understanding and experiencing the feelings and desires of oneself and others and consisted of understanding empathic resonation. The 4th was for expressing concretely and positively through the “request” of the four components of NVC, and the participants practiced empathic expressions. The 5th session was for listening empathically and expressing gratitude, while focusing on the other’s feelings and needs, and participants practiced perceiving empathy for others. The 6th session was for giving and receiving empathic communications and feedback with the other person by applying the components of NVC, and the cycle of empathy was to be achieved. This empathy process undergoes a sequence of stages to form “interpersonal empathy”, that includes cognitive, emotional, and communicative components. This study aimed to improve the participants’ self-esteem, empathic ability, interpersonal relationships, and communication competency. 

### 2.4. Data Collection and Intervention of the Program 

In this study, a pre-survey was conducted by a research assistant one week before applying the NVC-based empathy education program. A post-survey was conducted one week after applying the program to the participants. The personnel who participated in the study were 2 researchers (educators), and 1 research assistant who participated in the pre- and post-surveys. The researchers developed the program, and provided nonviolent communication education to the study participants. The research assistant was required to complete IRB education as a preparation for research, and was educated on the contents of the questionnaire and collection methods for data collection.

From 30 October to 12 December 2019, the experimental group was divided into two groups of 16 participants and underwent the NVC-based empathy education program in a total of six sessions. Each session was conducted for 120 min (2 h). The program was conducted using educational methods such as lectures, group activities, role play, emotion card activities, and video-clip-watching, according to the topic. After each session, the participants were given time to summarize learnings from the session by writing a reflection journal and sharing feedback with each other. In addition, participants received homework so that they could practice NVC in their daily life, and homework was checked at the next session. 

The lectures on the four components of NVC were conducted using PPT and video materials. Various examples and pictures were presented in the activity sheets for the participants to understand NVC’s principles and apply them in their daily lives. Regarding case-based group activities, teams of four participants each were formed and practiced NVC role-playing according to examples described in the activity sheets or individual cases. After group activities, they had time to share feedback while presenting their feelings to all the participants. The main scenario situations in the sessions included conversations between friends, parents and children, colleague nurses, and patients and nurses. The participants had time to carefully reflect on their own and the others’ feelings by writing scenarios; moreover, they experienced solving communication problems in various situations by directly expressing the components of NVC. Concerning emotion card activities, each participant was instructed to remind themselves of uncomfortable situations and to express emotions that were difficult to express in words using cards. Similarly, the participants could experience empathy for others naturally by listening to others’ stories and checking the feelings and needs of the other participants using emotion cards. After the end of each session, all participants were asked to write a reflection journal and to give presentations on the most impressive content (experience), opportunities to empathize with others, ways to view themselves positively, helpful ways to communicate, and helpful things in positive interpersonal relationships. After listening to each participant’s experiences, the educator provided feedback on what was good, what was subpar, and what needed to be improved.

After this study was completed, participants in the control group were provided with the same education as the experimental group if they wanted to be educated, and all participants were provided with portable mini cards containing a list of the principles, feelings, and requirements of NVC, and an educational booklet on how to use them. 

### 2.5. Data Analysis

Statistical analysis was performed using the SPSS 24.0 program. Pre-test homogeneity was analyzed using the χ^2^-test, Fisher’s Exact test, and independent t-test. All continuous variables (“age”, “self-esteem”, “empathic ability”, “interpersonal relationship”, “communicative competence”) were analyzed, after verifying their normal distribution and homoscedasticity using the Shapiro–Wilk normality test and Levene’s test for equality of variances, respectively. As a result, all continuous variables were fitted on the assumptions of population normality and homoscedasticity with a significance level of 0.05 or higher. Finally, hypothesis testing to determine the effects of the NVC-based empathy education program was performed using a two-group independent means t-test. 

### 2.6. Ethics and Informed Consent

This study was approved by the Institutional Review Board of Chonnam National University (1040198-190520-HR-044-04). Participants were informed of the study purpose, procedure, and period. Participation in this study was voluntary. Participants were free to withdraw from the study at any time. When the subjects filled in a survey questionnaire, it was considered agreement on informed consent. Participants’ anonymity and confidentiality were assured.

## 3. Results

### 3.1. Homogeneity Testing for the General Characteristics

The results of testing the homogeneity of the participants’ general characteristics in the experimental and control groups before the NVC-based empathy education program revealed no significant difference between the experimental and control groups, indicating that the two groups were homogeneous (Table 2).

By gender, 93.7% of the participants in the experimental group were female, and 83.3% were in the control group. By age, the proportion of those under 20 years in the experimental and control groups was 75.0% and 73.3%, respectively. Regarding religion, the proportions of those having no religion in the experimental and control groups were 65.6% and 63.3%, respectively. Regarding academic performance, the proportions of those with moderate, poor, and those with excellent in the experimental group were 59.4%, 34.3%, and 6.3%, respectively, while the proportions of those with moderate, those with poor, and those with excellent in the control group were 53.3%, 26.7%, and 20.0%, respectively. In terms of satisfaction with their major, the proportion of those reporting that they were satisfied with their major in the experimental and control groups were 56.3% and 46.7%, respectively, while the proportions of those reporting that they were moderately satisfied with their major in the experimental and control groups were 43.7%, and 53.3%, respectively. 

### 3.2. Homogeneity Testing for the Dependent Variables

The results of the homogeneity test for the dependent variables in the experimental and control groups before the NVC-based empathy education program found that there was no significant difference in self-esteem (t = 0.54, *p* = 0.595), empathic ability (t = 0.38, *p* = 0.705), interpersonal relationship (t = 0.30, *p* = 0.764) and communication competency (t = 0.72, *p* = 0.474), indicating that the two groups were homogeneous (Table 3).

### 3.3. Hypothesis Testing

All hypotheses were supported (Table 4).

(H1) The difference between post-test and pre-test self-esteem scores was 4.56 ± 3.98 points in the experimental group and −0.07 ± 4.98 points in the control group, indicating a statistically significant difference between the two groups (t = 4.06, *p* < 0.001).

(H2) The difference between the post-test and pre-test empathy scores was 22.28 ± 17.72 points in the experimental group and –1.13 ± 17.59 points in the control group, indicating a statistically significant difference between the two groups (t = 5.22, *p* < 0.001).

(H3) The difference between the post-test and pre-test interpersonal relationship scores was 14.59 ± 13.47 points in the experimental group and –3.00 ± 13.45 points in the control group, indicating a statistically significant difference between the two groups (t = 5.14, *p* < 0.001). 

(H4) The difference between post-test and pre-test communication competency scores was 9.53 ± 8.48 points in the experimental group and –0.87 ± 7.06 points in the control group, indicating a statistically significant difference between the two groups (t = 5.27, *p* < 0.001). 

## 4. Discussion

This study developed and applied an NVC-based empathy education program to nursing students and investigated its effects on self-esteem, empathy, interpersonal relationships, and communication competency in nursing students. 

This study found that the experimental group receiving the NVC-based empathy had improved self-esteem compared to the control group. This result is similar to a previous study [18] reporting that empathic self-awareness was improved in nursing students using an empathy program. In addition, a systematic review of NVC intervention studies [15] reported that participants demonstrated significantly improved self-esteem after NVS intervention. We theorize that, as the program progressed, the process of participants’ understanding, getting to know and empathize with others as individual human beings brought about positive changes in their self-esteem as a subjective evaluation of themselves. It is thought that the participants in this study explored their feelings and desires in given situations using emotion cards and exchanged support and positive feedback with each other through sharing in group activities, thereby changing their perception of themselves in a positive direction. 

This study demonstrated that the experimental group receiving the NVC-based empathy education had improved empathic ability compared to the control group. This result is similar to the results of a study by Jeong and Kim [28], reporting that an empathy education program for nursing students resulted in an improvement in taking the perspectives of other persons. In this study, the participants were educated to improve cognitive and emotional empathy involving the components of empathy from “empathic attention” to the “cycle of empathy.” In addition, repeated training and practice of paying attention to others’ situations and stories, as emphasized in NVC contents, and exploring the feelings and desires of oneself and others helped to improve empathy. A previous study [29], using NVC and meditation, found that participants had enhanced empathic ability to enable a deep understanding of others and compassion for others. This may be because participants’ practice of focusing on their feelings and needs and concentrating on their inner self using NVC might influence their awareness and acceptance of others. Suarez et al. [17] reported that in interviews with prisoners after applying NVC, they demonstrated the confidence to express their feelings, respond empathically to others, and communicated their needs well, confirming that NVC had a positive effect on improving empathy. Nosec et al. [30] described that an NVC program had a significant impact on empathy in nursing students and that the results of qualitative data analysis confirmed that the NVC program helped nursing students improve their understanding of themselves and interpersonal relationships. Empathy involves getting to know each other deeply while sharing feelings [9]. Thus, in the process of sharing feelings using NVC, the participants could understand and experience the others from their perspective, while breaking away from their egocentric thinking. This might influence enhanced empathic ability. In addition, most empathic intervention studies are inconsistent in terms of knowledge–attitude–behavior between empathy intervention and evaluation [31]. However, in this study, participants were given practice of the cognitive–emotional–behavioral aspects of empathy and confirmed the practical effects. Therefore, it can be expected that NVC-based empathy reinforcement training can be used as a very useful intervention.

This study showed that interpersonal relationships were improved in the experimental group receiving the NVC-based empathy education compared to the control group. This result is similar to the results of studies [18,28] using an empathy program in nursing students who have reported that interpersonal relationships and prosocial behaviors in nursing students were improved. Marlow et al. [16] reported that, because of using an NVC intervention in parolees admitted to a facility for substance abuse treatment, the ability to listen to others and interpersonal relationships were improved in parolees, which supports the results of this study. These results suggest that empathy has an essential effect on enhancing desirable and harmonious interpersonal relationships by increasing social interactions, thus increasing satisfaction with social interactions and achievements. Previous studies have emphasized the importance of empathic communication to build positive working relationships with co-workers and superiors [32]. In other words, to enhance interpersonal relationships, it is vital to improve cognitive, emotional, and communicative empathy involving understanding others, sharing emotions, and appropriate communication with others, to enhance interpersonal ability. Therefore, in this study, the participants were required to compare and observe their emotions and that of others through communicating and sharing feedback with them, examining their interpersonal relationships, and positively influencing each other. In addition, the participants were required to actively communicate with others using the NVC components and exchange feedback with each other, thereby strengthening a bond. In other words, the purpose of NVC is to change the quality of interpersonal relationships and can be achieved through communication to satisfy each other’s needs while respecting one’s feelings and needs and that of others. Thus, it is considered that the NVC program in this study was effective in enhancing interpersonal relationship. 

This study found that communication competency was improved in the experimental group receiving NVC-based empathy education compared to the control group. This result is similar to the results of prior studies [5,18,33] reporting that communication competency was improved in nursing students. In addition, those studies were mainly related to communication training between nurses and patients in nursing situations. Communication competencies in nursing professionals are important attitudes and skills for effectively dealing with interpersonal relationships with patients and others that may occur in various medical settings. Thus, nursing students should acquire empathic communication competency that is applicable to interpersonal relationships with patients and other people. Therefore, the participants in this study were given opportunities to communicate with each other by using various interpersonal relationship cases that considered daily life and clinical situations so that they could learn applicable empathy expressions based on shared understanding and respect for humans in interpersonal conflicts. In other words, the participants were presented with various conflict situations, such as those between parents and children, between friends, between colleagues, and between nurses and patients, and were instructed to express appropriate empathy using NVC. We theorize that NVC was not just a communication skill, but helped to perceive and respect individuals’ feelings and needs, influence individuals’ psychological changes, and express empathy. In addition, the program helped the participants to feel confident that they could resolve conflicts amicably through communication and helped to improve intimacy and communication competency while recognizing the differences between them and the other participants through role-playing and exchanging constructive feedback with each other.

This study found that the NVC-based empathy education program effectively improved nursing students’ self-esteem, empathic ability, interpersonal relationships, and communication competency. This study developed an empathy education program for nursing students from a theoretical perspective and investigated its effects, thus contributing to building a body of humanistic nursing knowledge that realizes the purpose of human dignity. In addition, from a practical perspective, we expect that such NVC-based empathy education will enhance empathic ability and empathy-based communication competency in nursing students. This will help to create an efficient organizational culture based on positive interpersonal relationships and communication with patients, caregivers, and colleagues in future clinical practice. Finally, from an education perspective, it is expected to improve nursing students’ interpersonal relationships, thereby enhancing their adaptability to university life.

The limitations were that the study involved first-year nursing students at two universities located in one metropolitan city in South Korea; there is a limitation in broadly interpreting and applying the results of this study to all nursing students. In addition, because no follow-up evaluation of the participants after six months of the education program was made, the continuity of the effects of the education program could not be confirmed. Since empathy through NVC can be maintained through continuous education and personal practice, post-intervention meetings or follow-up programs are needed to determine the long-term effects of the NVC-based empathy education program. 

## 5. Conclusions

This study investigated the effects of a NVC-based empathy education program on nursing students’ self-esteem, empathic ability, interpersonal relationships, and communication competency. The results revealed that the scores for self-esteem, empathic ability, interpersonal relationships, and communication competency were significantly higher in the experimental group than in the control group. Based on this study’s findings, it is necessary to perform continuous and repetitive education that can help nursing students improve empathy-based interpersonal relationships and communication competency within the nursing curriculum. In addition, it is also necessary to verify the effects of NVC-based empathy education programs on nursing students by academic year and level.

## Figures and Tables

**Table 1:** Nonviolent communication empathy education program based on cyclical empathy model.

Session	NVC Stage	Contents	Element of Empathy
1	Understanding of observing the NVC	General overview of the program and each session Finding frequently used languages, behavior, and facial expressions Difference between evaluation and observation	Cognitive (Empathic attentional set)
2	Awareness of my feelings and expressing honestly	NVC card game: Understanding vocabulary to express feelings Guessing the feelings of others in various situations Understand the feelings of me and others	Cognitive- Affective (Empathic resonation)
3	Understanding the needs of me and others	Awareness of self-feelings and needs Connecting feelings and desires Imagine the feelings and needs of others	
4	Request in positive behavioral language	Role play: Request others while respecting each one’s feelings and needs Distinguish between requesting and compelling	Communicative (Expressed empathy)
5	Listening empathically Expressing gratitude	Listening with empathy while focusing on the other’s feelings and needs Expressing gratitude by applying the NVC factors	Affective Communicative (Received empathy)
6	Communicating with the other person empathically	Exploring obstacles of empathy Role play: Empathic communication practice according to the situation Summary of program contents Sharing of impressions and promises	Cognitive Affective Communicative (Cycle of empathy)

**Table 2:** Homogeneity of general characteristics between the control and experimental group (*N* = 62).

Characteristics	Experimental Group (*n* = 32)	Control Group (*n* = 30)	χ^2^	*p*
	*n* (%)	*n* (%)		
Gender			1.677 ^†^	0.249
Female	30 (93.7)	25 (83.3)		
Male	2 (6.3)	5 (16.7)		
Age(years)			0.022 ^†^	1.000
20 or under	24 (75.0)	22 (73.3)		
21 or above	8 (25.0)	8 (26.7)		
Religion			0.036 ^†^	1.000
Yes	11 (34.4)	11 (36.7)		
No	21 (65.6)	19 (63.3)		
Academic performance			2.669 ^†^	0.263
Excellent	2 (6.3)	6 (20.0)		
Moderate	19 (59.4)	16 (53.3)		
Poor	11 (34.3)	8 (26.7)		
Satisfaction of major			0.569	0.612
Satisfied	18 (56.3)	14 (46.7)		
Moderate	14 (43.7)	16 (53.3)		

Note: ^†^ Fisher’s exact probability test.

**Table 3:** Homogeneity of variables between the control and experimental group (*N* = 62).

Characteristics	Experimental Group (*n* = 32)	Control Group (*n* = 30)	*t*-Test	*p*
	M ± SD	M ± SD		
Self-esteem	28.34 ± 3.06	27.90 ± 3.47	0.54	0.595
Empathic ability	100.31 ± 11.68	99.17 ± 12.06	0.38	0.705
Interpersonal relationship	85.16 ± 8.50	84.50 ± 8.61	0.30	0.764
Communicative competence	52.03 ± 4.73	51.20 ± 4.33	0.72	0.474

Note: M = mean; SD = standard deviation

**Table 4:** Comparison of dependent variables between experimental group and control group (*N* = 62).

Variables	Experimental Group (*n* = 32)	Control Group (*n* = 30)	t
	M ± SD	M ± SD	
Self-esteem	4.56 ± 3.98	−0.07 ± 4.98	4.06 ***
Empathic ability	22.28 ± 17.72	−1.13 ± 17.59	5.22 ***
Interpersonal relationship	14.59 ± 13.47	−3.00 ± 13.45	5.14 ***
Communicative competence	9.53 ± 8.48	−0.87 ± 7.06	5.27 ***

Note: M = mean; SD = standard deviation; *** *p* < 0.001.

## Data Availability

The data sets generated for the present study will be available from the corresponding author upon request.
